# Geriatric patients' expectations of their physicians: findings from a tertiary care hospital in Pakistan

**DOI:** 10.1186/1472-6963-9-205

**Published:** 2009-11-13

**Authors:** Taimur Saleem, Umair Khalid, Waris Qidwai

**Affiliations:** 1 Medical College, The Aga Khan University, Stadium Road, Karachi 74800, Pakistan; 2 Department of Family Medicine, The Aga Khan University, Stadium Road, Karachi 74800, Pakistan

## Abstract

**Background:**

Geriatric health is a neglected and under-explored area internationally and in Pakistan. We aimed to ascertain the expectations of the geriatric patients from their physicians and the factors associated with patient satisfaction in this particular age bracket.

**Methods:**

A cross-sectional survey was carried out at a tertiary care teaching hospital in Karachi, Pakistan. Data collection was carried out via face-to-face interviews based on structured, pre-tested questionnaires. All consenting individuals aged 65 years or above were recruited into the study. Convenience sampling was used to draw the sample. The data was analyzed using SPSS version 16. Geriatric patient's expectations from physicians were elicited using a set of 11 questions that were graded on a scale of 1-3 where 1 = not important, 2 = important, 3 = very important.

**Results:**

Three hundred and eighty geriatric patients were interviewed. The response rate of this study was 89.8%. The mean age of the respondents was 73.4 ± 6.8 years. Two hundred and forty eight respondents (65.3%) were female. Diabetes mellitus (53.7%), hypertension (59.5%), arthritis (40.5%) and renal disease (32.1%) were common ailments among geriatric patients. More than 50% of the patients were visiting their physicians once every two to three months. Discussing treatment options and letting patients make the final decision (79.2%), prescribing minimum possible medications (84.5%), physician's holistic knowledge about the spectrum of care issues for geriatric patients (79.2%), being given a realistic but optimistic picture of future health by physicians (85.5%) were ranked as very important expectations by patients from their physicians. Cumulative household income (p = 0.005), most important health complaint (p = 0.01) and frequency of experiencing health complaint (p < 0.001) emerged as independent predictors of the satisfaction of geriatric patients from care provided by physicians.

**Conclusion:**

We have documented the expectations of the geriatric patients from their physicians in a developing country. Physicians belonging to all disciplines should keep these expectations in mind during clinical encounters with geriatric patients.

## Background

Geriatric population is a rapidly growing age bracket globally. The majority of this elderly population (60% of the 580 million elderly people globally) is living in the developing countries. By 2020, this value will increase to 70% of the total elderly population [1]. It is estimated that between 2000 and 2050, the proportion of individuals above the age of 65 will more than double from 6.9% to 16.4% [2]. Pakistan, currently the sixth most populous country in the world, has an estimated geriatric population of around 7 million [3]. However, this segment of the society is not receiving its due share of health services despite facing a myriad of medical problems [4,5]. Issues such as paucity of appropriate retirement benefits and pensions [6] as well as the evanescence of the traditional joint family system in Pakistan are important considerations for the geriatric population [7].

The universe of the health care delivery system centers on patients. It is, therefore, important to procure their input regarding health services and their expectations from such services. This may improve the level of patient satisfaction [8] and also favorably impact the effectiveness of the consultation [9].

A thorough MEDLINE search revealed that although literature regarding the expectations of geriatric patients from healthcare services is available, this literature is geared towards very specific aspects of geriatric healthcare; for example expectations towards implantable cardioverter-defibrillators [10], day-care rehabilitation services [11], social services [12], various surgical services such as vascular, cardiac and spinal surgery [13-15], percutaneous transluminal coronary angioplasty [16] and nursing homes [17]. At other times, the available literature has focused on specific groups of geriatric patients; for example stroke patients [18], hypertensive patients [19], dialysis patients [20], patients recovering from surgery [21] or institutionalized patients [22]. Still further, research has focused on healthcare professionals other than physicians such as nurses [23]. Our literature search revealed a dearth of information with regards to the general expectations of geriatric patients from physicians generally and especially in Pakistan despite a sizeable population being 65 years and above. Therefore, both medical and mental health of geriatric patients runs the risk of under-treatment and under-recognition.

According to World Health Organization (WHO), "health is a state of complete physical, mental and social well-being and not merely the absence of disease or infirmity" [24]. Caring for the elderly not only comprises medical and technical aspects but the psychological and social dimensions of health as well [25]. Therefore, physicians should be cognizant of the complete spectrum of health needs of geriatric patients. We carried out a survey among a selected geriatric patient population in Karachi, the largest metropolitan of Pakistan, to ascertain their general expectations from physicians. We also aimed to determine the factors associated with patient satisfaction among this particular age demographic. Physicians can assimilate this knowledge into their practice to make the care provided more meaningful and effective [26].

## Methods

### Study design and site

We conducted a cross-sectional survey to gauge the expectations of the geriatric patients at the Community Health Centre (CHC) at The Aga Khan University Hospital (AKUH), Karachi, Pakistan. AKUH is a tertiary level teaching facility in the private sector and caters to the medical needs of a large majority of patients coming from all over Karachi as well as from other parts of Pakistan. Consenting geriatric patients encountered in the waiting area of the CHC clinics were recruited into the survey over a one month period. CHC clinics were chosen because they are attended by a large number of people from all walks of life and all ages. These clinics cater to patients from almost all socioeconomic classes. Another important consideration in selection of CHC was that it not only runs family medicine clinics, but also provides specialist services to a number of patients. Therefore, a diverse assortment of patients from all backgrounds was expected to be encountered there.

### Study sample and data collection

We required a sample size of 385 to fulfill our objectives at 95% confidence interval, and 5% sample error. This sample size was calculated assuming a 50% variance due to paucity of prior studies on the subject. It was further adjusted for a 10% non-response rate; bringing the total sample size to 420 respondents. Convenience sampling was used to draw the sample. All consenting individuals aged 65 years and above and visiting the CHC between 10 am to 4 pm were interviewed. Information was collected using face-to-face interviews based on a structured, pre-tested questionnaire. We anticipated the potential problem that elderly patients may experience in filling the questionnaires; therefore they were interviewed rather than being asked to fill in questionnaires by hand. People who could not comprehend the questions asked by the interviewers, either due to a language barrier or severe cognitive impairment were excluded from the survey. The questionnaire was thoroughly discussed among the interviewers before data collection to decrease interview bias.

### Questionnaire [see Additional File 1]

#### a. Development

The initial questionnaire was developed on the basis of literature search and prior experience of investigators. In addition, colleagues, peers, patients and their attendants were explained about the study and its objectives in simple and clear language and their informal input was sought. Their suggestions were incorporated into the questionnaire. The questionnaire so prepared was then formally pre-tested on 25 respondents of the target population. The pre-testing enabled investigators to ascertain the conceptual deficiencies as well as the feasibility of the administration of the data collection tool. Ambiguities and duplications in the questionnaire were removed and clarity was introduced. Our final questionnaire was a result of these efforts. The results of the pre-testing were not included in the final analysis of the data.

#### b. Efforts to reduce bias

A meeting of the investigators was held prior to data collection in order to maintain uniformity in the manner the face-to-face interviews were conducted; hence reducing chances of interviewer's bias in the study.

#### c. Linguistic validation of questionnaire

The questionnaire was initially prepared in English; then translated into Urdu, the national language of Pakistan, for the convenience of the respondents. The questionnaire was also back-translated into English by two non-medical personnel to check for any paraphrasing errors. This provided linguistic validation for the questionnaire.

#### d. Contents of questionnaire

The questionnaire consisted of four sections.

**I **- Section one dealt with the socio-demographic details of the respondents.

**II **- Section two briefly enquired about the medical ailments and symptomatology of participants.

**III **- Section three contained a set of questions aimed at eliciting the geriatric patient's expectations from his physician using a scale of 1-3 where 1 = not important, 2 = important, 3 = very important.

**IV **- Section four enquired about the frequency of visits of geriatric patients to the doctor along with overall satisfaction with treatment provided.

### Data management and statistical analysis

Data was entered twice and validated using Windows Statistical Package for Social Sciences (SPSS) version 16.0. It was cleaned for invalid and out of range values, missing values and duplicate ID numbers. The data was then analyzed using the same software. As part of descriptive statistics, mean, median, mode and standard deviations were used to express the continuous data while proportions were calculated for categorical data. Associations were assessed using Chi-square test and Fisher's exact test where appropriate. For all analysis performed for the data set, the level of significance (α) was set as 0.05. Variables with a significant p-value in chi-square testing were further subjected to a multiple logistic regression analysis to determine factors which are independent predictors of the satisfaction of geriatric patients from the care provided. All odds ratios were recorded with a 95% confidence interval. As s further step, we also determined the goodness of fit of the model to measure how well it described our response variable by using Hosmer-Lemeshow test.

### Ethical Considerations

Informed consent for participation in the survey was obtained from the respondents of the survey; this consent was either written or verbal. Strict confidentiality of the information was maintained throughout the process of data collection, entry and analysis. All efforts were made in this study to fulfill the ethical considerations in accordance with the 'Ethical principles for medical research involving human subjects' of Helsinki Declaration [27]. The confidentiality of each participant was strictly ensured throughout the project. The study was approved by the Ethical Review Committee (ERC) at the Department of Community Health Sciences, Aga Khan University Hospital.

## Results

A total of 423 geriatric patients were approached for this survey. Forty three patients declined to participate while 380 patients completed the full interviews. Therefore, the response rate of this study was 89.8%.

### Socio-demographic characteristics

The mean age of the respondents was 73.4 ± 6.8 years. Two hundred and forty eight respondents (65.3%) were female. Diabetes mellitus (53.7%), hypertension (59.5%), arthritis (40.5%), renal disease (32.1%) and ischemic heart disease (25.3%) were common ailments afflicting the geriatric population in our sample. However, cancer (5%) and cerebrovascular accident (11.1%) were less common. When enquired about their most important health complaints, geriatric patients reported urinary incontinence (22.4%), myalgias or arthralgias (20.3%), and memory loss (13.9%) as important health complaints. With regards to the frequency of visits to the doctor, more than 50% of the patients were visiting their physicians at least once every two to three months. A total of 83 participants were current smokers. Out of these 83 individuals, 48 (57.8%) were males and 35 (42.2%) were females. The association between current smoking status and gender was statistically significant (p < 0.001). The baseline characteristics of our sample are shown in table 1.

**Table 1:** Baseline characteristics of geriatric patients in our sample

Baseline Characteristics	Frequency (n = 380)	%
**Age in years**		
- 65-70	160	42.1
- 71-75	71	18.7
- 76-80	76	20
- 81-85	58	15.3
- 85-90	15	3.9

**Gender**		
-Male	132	34.7
-Female	248	65.3

**Marital Status**		
-Single	52	13.7
-Married (and living with spouse)	179	47.1
-Divorced or Separated	57	15
-Widowed	92	24.2

**Education**		
-Illiterate/Can read or write name only	56	14.7
-Up to Primary *	58	15.3
-Up to Secondary **	70	18.4
-Up to Higher Secondary ***	108	28.4
-Graduation or Post graduation	88	23.2

**Cumulative household income in Pakistani Rupees ^**		
**- <**5000	51	13.4
-5000-10,000	56	14.7
- > 10,000-25,000	98	25.8
- > 25,000-50,000	121	31.8
- > 50,000-100,000	36	9.5
- > 100,000	18	4.7

**Frequency of experiencing health complaints**		
- Daily	59	15.5
- 2-5 times a week	126	33.2
- Once a week	85	22.4
- Twice or thrice a month	50	13.2
- Once a month	17	4.5
- Less frequently than once a month	43	11.3

**Frequency of Visits to the Doctor**		
- Weekly	18	4.7
- Every 2-4 weeks	40	10.5
- Every 2-3 months	239	62.9
- Every 6 months	70	18.4
- Annually	13	3.4

**Currently Smoking**		
Yes	83	21.8
No	297	78.2

* Upto grade 5, ** Upto grade 10, *** Upto grade 12, ^ 1 US $: 83.23 PK Rupees

### Geriatric patients' expectations from their physicians

Table 2 shows the response of the geriatric patients when asked about their expectations from their physicians in terms of various aspects of care. This was done by providing statements and asking the geriatric patients to rank them on a scale of 1 to 3 with 1 being not important and 3 being very important. For the geriatrics patients, aspects such as explaining the nature of the disease in clear and simple language (60%), discussing all available treatment options and letting patients make the final decision (79.2%), prescribing minimum possible medications (84.5%), telling patients how long the illness will last and how many follow-ups are required (74.2%), physician's holistic knowledge about the spectrum of care issues for geriatric patients (79.2%), being given a realistic but optimistic picture of future health by their physicians (85.5%) and use of both verbal and non-verbal gestures by their doctors to comfort them (60.3%) were ranked as very important expectations.

**Table 2:** Geriatric patients' expectations from their physicians

Expectations from physicians in terms of	n = 380 (%)		
	
	Not Important	Important	Very Important
1. Explaining the nature of the disease in clear and simple language	59 (15.5)	93 (24.5)	228 (60)
2. Letting you talk freely about your illness and problems	48 (12.6)	196 (51.6) (35.8)	136
3. Involving your family and friends in the consultation with your permission	161 (42.4)	119 (31.3) (26.3)	100
4. Discussing all available treatment options and letting you make the final decision	28 (7.4)	51 (13.4) (79.2)	301
5. Prescribing minimum possible Medicines	21 (5.5)	38 (10) (84.5)	321
6. Informing you about any side effects of treatment prescribed	37 (9.7)	202 (53.2) (37.1)	141
7. Telling you how long the illness will last and the number of follow-ups needed	35 (9.2)	63 (16.6) (74.2)	282
8. Not refusing to treat you on the grounds of age	106 (27.9)	77 (20.3) (51.8)	197
9. Knowing about the complete spectrum of care issues for elderly patients	31 (8.2)	48 (12.6) (79.2)	301
10. Giving you a realistic but optimistic picture of your health and future	22 (5.8)	32 (8.4) (85.8)	326
11. Using both non-verbal and verbal gestures to comfort you when needed	18 (4.7)	133 (35)	229 (60.3)

### Satisfaction with care provided

Two hundred and forty five patients (64.5%) patients were fully satisfied that the care being provided to them by physicians was appropriate for their age demographic. However, 135 patients (35.5%) were not fully satisfied with the care provided. We computed the association between various factors and the satisfaction of the geriatric patients from physicians as shown in table 3. The satisfaction with the care provided by physician was significantly association with the age of the geriatric patients (p = 0.009), most important health complaint (p = 0.006), cumulative household income (p = 0.005), frequency of experiencing symptoms (p < 0.001) and frequency of visits to physician (p = 0.01).

**Table 3:** Association of various factors with the satisfaction of geriatric patients from care provided by their physicians

Variables	Satisfaction with the level of care provided (n = 380)	
	
	Yes	No
**Age in years (p = 0.009)**		
- 65-70	106	54
- 71-75	46	25
-76-80	41	35
-81-85	46	12
- 86-90	6	9

**Gender (p = 0.21)**		
- Male	81	51
- Female	164	84

**Marital status (p = 0.72)**		
- Single	35	17
- Married (and living with spouse)	110	69
- Divorced/separated	38	19
- Widowed	62	30

**Education (p = 0.22)**		
- Illiterate or can read and write name only	31	25
- Up to primary *	40	18
- Up to secondary **	43	27
- Up to higher secondary ***	67	41
- Graduate or post graduate studies	64	24

**Cumulative household income in Pk rupees ^ (p = 0.005)**		
- < 5,000	35	16
- 5,000-10,000	44	12
- > 10,000-25,000	70	28
- > 25,000-50,000	70	51
- > 50,000-100,000	16	20
- > 100,000	10	8

**Most important health complaint (p = 0.006)**		
- Myalgias/arthralgias	40	37
- Dyspnea	4	6
- Nausea/vomiting	8	3
- Insomnia	15	13
-Anorexia	13	9
- Bed sores	9	4
- Urinary incontinence	60	25
- Urinary retention	12	5
- Fecal incontinence	25	3
- Constipation	29	7
- Memory loss	30	23

**Frequency of experiencing health complaints (p < 0.001)**		
- Daily	30	29
- 2-5 times a week	78	48
- Once a week	53	32
- 2-3 times a month	44	6
- Once a month	16	1
- Less frequently than once a month	24	19

**Frequency of visits to the doctor (p = 0.01)**		
- Weekly	7	11
- Once every 2-4 weeks	28	12
- Once every 2-3 months	162	77
- Once every 6 months	44	26
- Annually	4	9

* Upto grade 5, ** Upto grade 10, *** Upto grade 12, ^ 1 US $: 83.23 PK Rupees

These variables were then further subjected to multiple logistic regression analysis to ascertain the independent predictors of the satisfaction of geriatric patients from physicians while adjusting for confounders (table 4). Factors such as cumulative household income (p = 0.005), most important health complaint (p = 0.01) and frequency of experiencing health complaints (p < 0.001) emerged as independent predictors of the satisfaction of geriatric patients with the care provided. Frequency of visits to the doctor (p = 0.06) and age of geriatric patients (p = 0.073) were found to be marginally associated with the satisfaction of geriatric patients from the care provided.

**Table 4:** Multiple logistic regression analysis showing independent predictors of satisfaction of geriatric patients with the care provided

Variables	Adjusted OR*	95% CI**
**Age in years (p = 0.073)**		
- 65-70	1	-
- 71-75	0.89	0.49-1.59
-76-80	0.69	0.39-1.22
-81-85	1.16	0.6-2.23
- 86-90	0.24	0.07-0.74

**Cumulative household income in Pk rupees ^ (p = 0.005)**		
- < 5,000	1	-
- 5,000-10,000	1.68	0.7-4
- > 10,000-25,000	1.14	0.54-2.38
- > 25,000-50,000	0.63	0.31-1.25
- > 50,000-100,000	0.37	0.15-0.88
- > 100,000	0.57	0.19-0.72

**Most important health complaint (p = 0.01)**		
- Myalgias/arthralgias	1	-
- Dyspnea	0.93	0.25-3.55
- Nausea/vomiting	4.16	0.84-20.5
- Insomnia	3.39	1.24-9.3
-Anorexia	1.62	0.61-4.3
- Bed sores	2.08	0.59-7.3
- Urinary incontinence	1.98	0.95-3.36
- Urinary retention	0.5	0.17-1.5
- Fecal incontinence	1.43	0.59-3.44
- Constipation		
	2.1	0.91-4.86
- Memory loss		
	3.5	1.58-7.86

**Frequency of experiencing health complaints (p < 0.001)**		
- Daily	1	-
- 2-5 times a week	1.57	0.84-2.93
- Once a week	1.60	0.81-3.13
- 2-3 times a month	7.09	2.62-19.15
- Once a month	15.47	1.92-124.26
- Less frequently than once a month	1.22	0.55-2.68

**Frequency of visits to the doctor (p = 0.06)**		
	1	-
- Weekly	1.42	0.45-4.41
- Once every 2-4 weeks	3.64	1.35-9.78
- Once every 2-3 months	2.35	0.81-6.81
- Once every 6 months	5.23	1.05-25.96
- Annually		

* Odd's ratio, ** Confidence interval, ^ 1 US $: 83.23 PK Rupees

The Hosmer-Lemeshow test of goodness of fit was not significant (χ^2 ^= 6.26, df = 8; significance = 0.596); thus the model fits the data. The Nagelkerke R^2^, an adjusted version of the Cox & Snell R^2^, was 0.183; providing a measure of effect size. The total percentage of the correct classification of the model was 65.3%. Furthermore, the discrimination of the model was assessed using the area under the receiver operating characteristic curve (AUROC). The c-statistic, adjudged by AUROC, was 0.73 ± 0.026 [95% confidence interval: 0.68 - 0.78).

## Discussion

The age at which individuals are considered "elderly" or "geriatric" varies from study to study. However, by convention, age 65 is usually taken as the minimum age, which we also followed [28]. In our study, most of the participants were below the age of 70 owing to a relatively lower life expectancy in our region. We can explain this on the basis of the limited access of the elderly population to the healthcare services; probably poor risk factor control of chronic diseases and difference in genetic makeup of the population in developing countries as compared to the developed world. We assessed the relationship between the socio-demographic variables and the patient satisfaction with health care. We found that age, most important health complaint and frequency of symptoms experienced had a significant relationship with their level of satisfaction with health care. A previous study from Pakistan has found a statistically significant association between depression in the elderly and satisfaction with health care services [29].

With advancing age, morbidity patterns evolve and transition. The incidence of chronic diseases and psychological ailments increases and rehabilitative support becomes essential [1]. In our study, diabetes and hypertension was present in more than half the patients, and the influence of these co-morbid conditions could be enormous in affecting the overall health and functioning of the geriatric population. A comparison with a Western study assessing the impact of different diseases in geriatric population showed that although the prevalence of hypertension was comparable, diabetes mellitus was a much bigger problem in our region [30].

A better understanding of the processes through which elderly individuals perceive, evaluate, and act on symptoms will enable physicians to respond more appropriately to the needs of older patients. In the same vein, we tried to decipher the expectations of the elderly population by asking specific questions. A significant proportion (79.2%) of the participants in our study strongly felt that the physician should discuss all available treatment options thoroughly and involve them in decision making. This is in agreement with previous studies in which the patients expected the physicians to understand the requests that they have regarding how they hope to be helped, and their treatment plan being an agreement between them and their physicians rather than being a unilateral dominion. This bilateral agreement between the physicians and the patients has been termed "concordance" [8,9]. This idea is further reinforced in our study in which the participants mentioned that both the patients and their family members should be allowed to talk freely about the illness. The findings in our study also support the notion of the transition of medical practice from a paternalistic model to an autonomous model in developing countries whereby geriatric patients expressed favorable views towards empowerment in the decision making process. In the paternalistic model, the physician makes the decision because it is assumed that he knows what is best for the patient. On the other hand, an autonomous model gives the patients the chance to participate in the decision making process of their care [31].

The participants of our study were keen to be informed about the nature of the disease in clear and simple language. Discussing biomedical issues is more important for the patients than discussing psychological issues. The patients want to hear about the cause and seriousness of their symptoms and about test results in clear and simple language [32]. Hence, it is imperative for the physicians to educate the patients thoroughly regarding the disease. At the same, the patients feel that they should be given a realistic but optimistic picture regarding their health status. This is because unmet expectations adversely affect the doctor-physician relationship. Physician's non-fulfillment of patient's requests plays a significant role in patient's beliefs that physicians did not meet their expectations for care [33].

### Strengths and limitations

This survey provides an insight into the expectations of geriatric patients from their physicians. It was conducted in a developing country with a burgeoning elderly population. As such, this geographical region and this topic are both generally under-represented in medical literature and this document would therefore be an important addition to the armamentarium of medical knowledge. In addition, our questionnaire may be helpful to other investigators who wish to gauge similar parameters in the future.

At the same time, we also acknowledge the following limitations of our study. These limitations must be considered before generalizing the findings of our study to other populations. We aimed to conduct a survey in a selected geriatric patient population at a tertiary care teaching hospital in Karachi, Pakistan. We used convenience sampling to draw this sample. This method of sampling is inherently inferior to probability sampling in its representativeness of the population, and this limits the external validity of the study. Also, this survey was conducted at an ambulatory clinic centre of a high-end hospital of a large cosmopolitan city. As such, individuals of educated, urban backgrounds or belonging to high income bracket would be expected to be encountered there. However, as mentioned before, CHC is unique in that it receives people from all socioeconomic classes and educational backgrounds and this aspect helped in offsetting this particular limitation of the study. Additionally, interviewers met beforehand to discuss the questionnaire; this measure was intended to reduce the interviewer's bias in the survey. However we acknowledge that the use of interviewing technique for data collection may have introduced some bias in the process although all efforts were made to minimize it.

## Conclusion

We have documented the expectations of the geriatric patients from their physicians in a selected population of geriatric patients encountered at an outpatient centre of a developing country. This is an important step in the improvement of future geriatric-physician interactions. Physicians should keep these expectations in mind during their clinical encounters with geriatric patients. Further targeted research on this issue should be carried out in the future to improve the level of care provided to geriatric patients. Specialized geriatric clinics should also be set up utilizing these results.

There is no doubt that geriatrics will rapidly evolve as a medical discipline in the coming years. The goals of geriatric medicine will, however, only be accomplished if geriatricians and their partners work in a system that is designed to provide high-quality and efficient care; a system that recognizes the value and understands the unique nature of the problems of geriatric patients. If the expectations of the patients are adequately met, then the level of satisfaction with health care will surely increase in parallel. Our study is an important step in that direction.

## Abbreviations

CHC: Community Health Center; AKUH: Aga Khan University Hospital; WHO: World Health Organization; SPSS: Statistical Package for Social Sciences

## Competing interests

The authors declare that they have no competing interests.

## Authors' contributions

TS was involved in study conception and design, data collection, entry, analysis, revision, editing and manuscript writing. UK collected and entered data, and participated in writing the manuscript. WQ was involved in study conception, manuscript writing, revision, editing and overall supervision. All authors have read and approved the final manuscript.

## Pre-publication history

The pre-publication history for this paper can be accessed here:

http://www.biomedcentral.com/1472-6963/9/205/prepub

## Supplementary Material

Additional file 1 **Questionnaire of Survey**. Face-to-face interviews were carried out on the basis of this questionnaire to gauge the expectations of geriatric patients from their physicians. Click here for file
