# Supplementary material for: Geriatric patients' expectations of their physicians: findings from a tertiary care hospital in Pakistan
Source: BMC Health Serv Res. 2009 Nov 13;9:205. doi: 10.1186/1472-6963-9-205 (PMC2780408; doi:10.1186/1472-6963-9-205)
Supplement: Additional file 1 — Questionnaire of Survey. Face-to-face interviews were carried out on the basis of this questionnaire to gauge the expectations of geriatric patients from their physicians. [file 1472-6963-9-205-S1.doc]

**ID number**: _____________________________

**Questionnaire**

**Expectations of geriatric patients from their physicians**

***Instructions to be given to the Respondents by the Interviewers before the start of Interview:***

*This is a* ***questionnaire based interview****, and will take approximately* ***10-12******minutes*** *to complete. Respondents should choose the option they deem most appropriate for each question. Some questions may require them to choose more than one option. Thank you*

**Section-I**

**1.1** Age ⁪⁪⁪

**1.2** Sex 1.Male ⁪ 2.Female ⁪

**1.3** Marital Status:

1.Single ⁪ 2.Married ⁪ 3.Divorced ⁪ 4.Widowed/widower ⁪ 5.Separated ⁪

**1.4** Education:

1.Illiterate ⁪ 2.Can read/write ⁪ 3.Primary ⁪ 4.Secondary ⁪ 5.Intermediate ⁪ 6.Graduate ⁪ 7.Postgraduate ⁪ 8.Diploma ⁪

**1.5** Religion:

1. Islam ⁪ 2.Christianity ⁪ 3.Hindu ⁪

4. Other (Please Specify) ____________________

**1.6** Household monthly income:

1. < Rs. 5000 ⁪ 2. Rs. 5000 – 10, 000 ⁪ 3. > Rs. 10,000 – 25,000 ⁪

4. > Rs. 25,000 – 50,000 ⁪ 5. > Rs. 50,000 – 100,000 ⁪

6. > Rs. 100,000 ⁪

**1.7** Do you currently smoke?

1. Yes ⁪ 2. No ⁪

If yes, please mention how much and since how long:

If you answered in negative, then have you ever smoked before?

1. Yes ⁪ 2. No ⁪

If yes, then please mention how much and for what duration of time?

**Section-II**

**2.1** Do you have any medical illness?

1. Diabetes ⁪ 2. Hypertension ⁪ 3. Renal Disease ⁪

4. Liver disease ⁪ 5. IHD/CHF ⁪ 6. Arthritis ⁪

7. Psychiatric illness ⁪ (Details: ____________________)

8. Cancer ⁪ 9. COPD ⁪ 10. CVA ⁪

8. Others (Please Specify)­­­­­­­­­­­­­­______________________

**2.2** Which disease symptoms do you currently experience:

1. Body pain ⁪ 2. Dyspnea ⁪ 3. Nausea/vomiting ⁪

4. Insomnia ⁪ 5. Loss of appetite ⁪ 6.Bedsores ⁪

7. Urinary incontinence ⁪ 8. Fecal incontinence ⁪

**Section-III**

**3.1** Please answer the following questions by grading them according to:

1(Not important) 2(Important) 3(Very important) 4 (Don’t know)

What are your expectations from the physicians in terms of?

| 1 | 2 | 3 | 4 |
| --- | --- | --- | --- |
| 1 | 2 | 3 | 4 |
| 1 | 2 | 3 | 4 |
| 1 | 2 | 3 | 4 |
| 1 | 2 | 3 | 4 |
| 1 | 2 | 3 | 4 |
| 1 | 2 | 3 | 4 |

1. Explaining the nature of the disease in clear and simple language

2. Letting you freely talk about your illness and problems

3. Involving your family and friends in the consultation with your permission

4. Discussing all available treatment options and letting you make the final decision

5. Prescribing minimum possible medicines

6. Informing you about any side effects of treatment prescribed

7. Telling you how long the illness will last and the number of follow-ups needed

8. Not refusing to treat you on the grounds of age

| 1 | 2 | 3 | 4 |
| --- | --- | --- | --- |
| 1 | 2 | 3 | 4 |
| 1 | 2 | 3 | 4 |
| 1 | 2 | 3 | 4 |

9. Knowing about the complete spectrum of care issues for elderly patients

10. Giving you a realistic but optimistic picture of your health and future

11. Using both non-verbal and verbal gestures to comfort you when needed

**Section-IV**

**4.1** What is your frequency of visits to the doctor:

1. Weekly ⁪ 2. Every 2-4 weeks ⁪ 3. Every 2-3 months ⁪

4. 6 monthly ⁪ 5. Annually ⁪

**4.2** Are you satisfied with the treatment being provided by your physician:

1. Yes ⁪ 2. No ⁪

***Thank you for your time.***

***Your suggestions on the questionnaire are most welcome.***
